# Supplementary material for: Augmented Ouabain-Induced Vascular Response Reduces Cardiac Efficiency in Mice with Migraine-Associated Mutation in the Na+, K+-ATPase α2-Isoform
Source: Biomedicines. 2023 Jan 25;11(2):344. doi: 10.3390/biomedicines11020344 (PMC9953359; doi:10.3390/biomedicines11020344)
Supplement: Supplementary file 1 [file biomedicines-11-00344-s001.zip › biomedicines-2128057-supplementary-figure.pdf]

Supplementary Figure 1

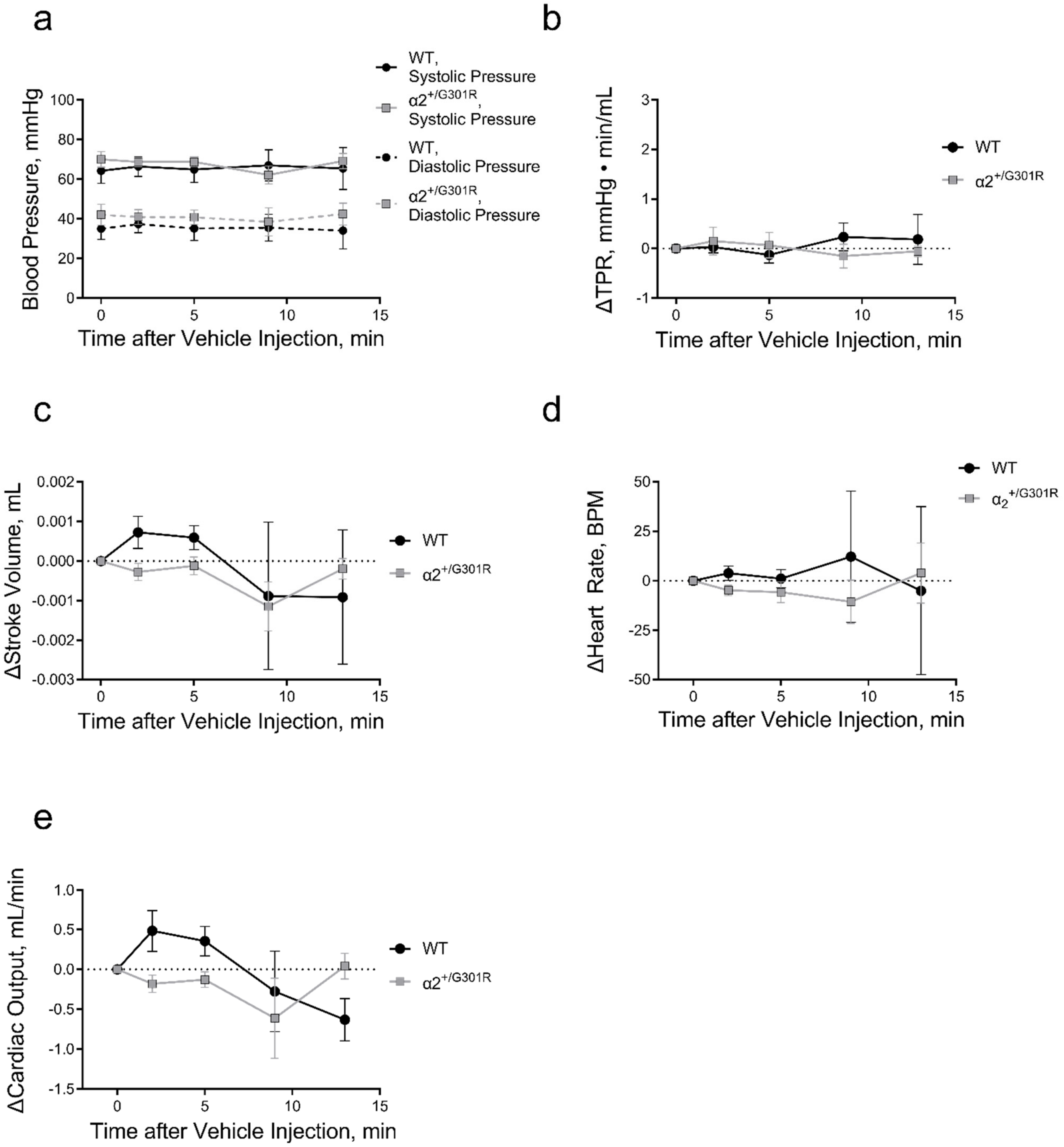

Figure S1. Vehicle administration (0.9% NaCl) did not change major cardiovascular variables in wild-type (WT) and $\alpha_2^{+/G301R}$ mice. (**a**) Blood pressure, (**b**) total peripheral resistance (TPR), (**c**) stroke volume, (**d**) heart rate, and (**e**) cardiac output were all unaffected by vehicle administration in both $\alpha_2^{+/G301R}$ and WT mice. Data were compared with two-way repeated measures ANOVA. $n = 4$.

Supplementary Figure 2

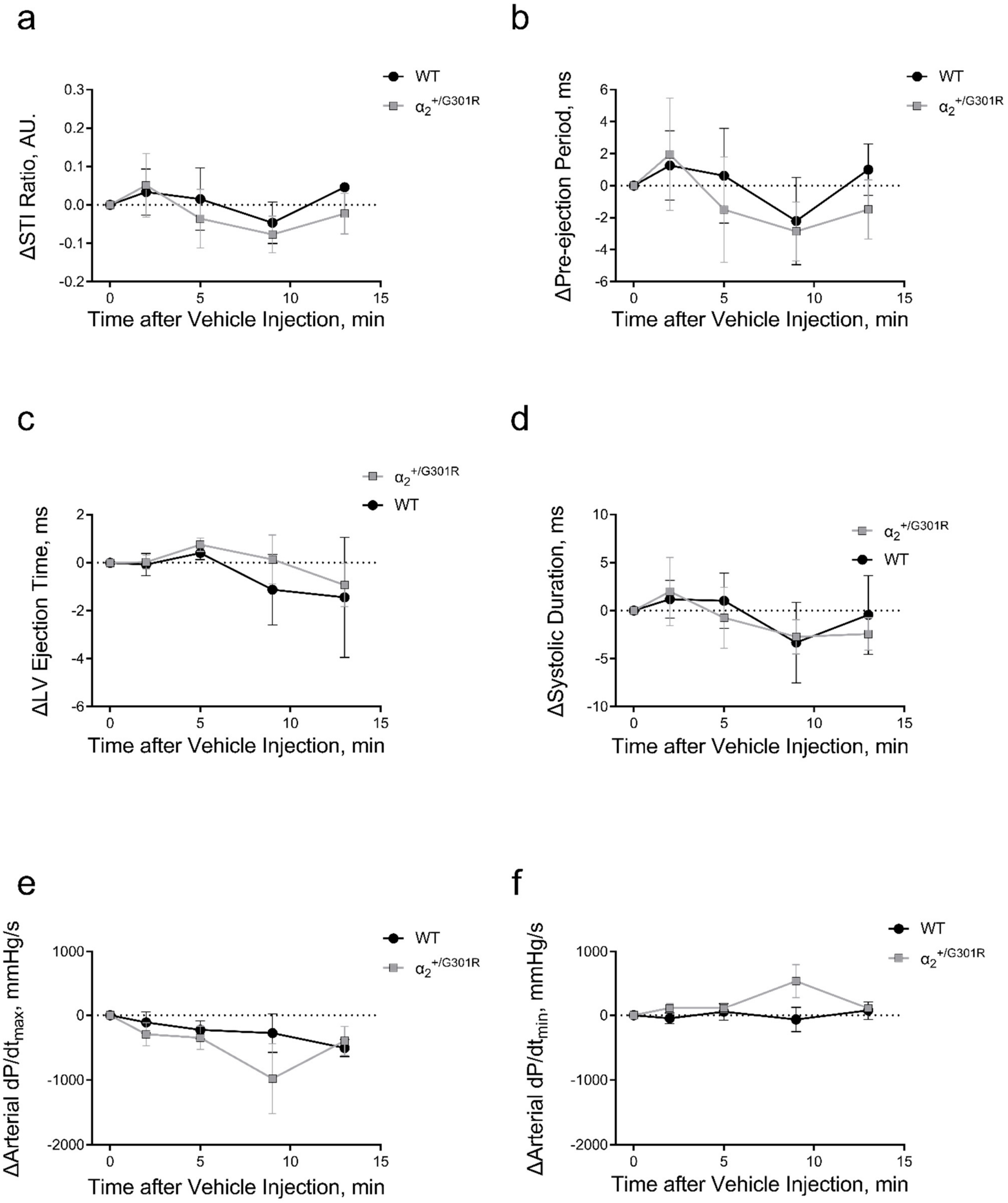

Figure S2. Vehicle administration (0.9% NaCl) did not alter cardiac function in both wild-type (WT) isoform-specific $\alpha_2^{+/G301R}$ mice. Following vehicle injection, (**a**) the ratio of systolic time intervals (STI Ratio), (**b**) pre-ejection period (**c**) left ventricular ejection time (LV ejection time), (**d**) systolic duration, (**e**) arterial dP/dt_{max}, and (**f**) arterial dP/dt_{min} were unchanged in both WT and $\alpha_2^{+/G301R}$ mice. Data were compared with two-way repeated measures ANOVA. *n* = 3-4.

Supplementary Figure 3

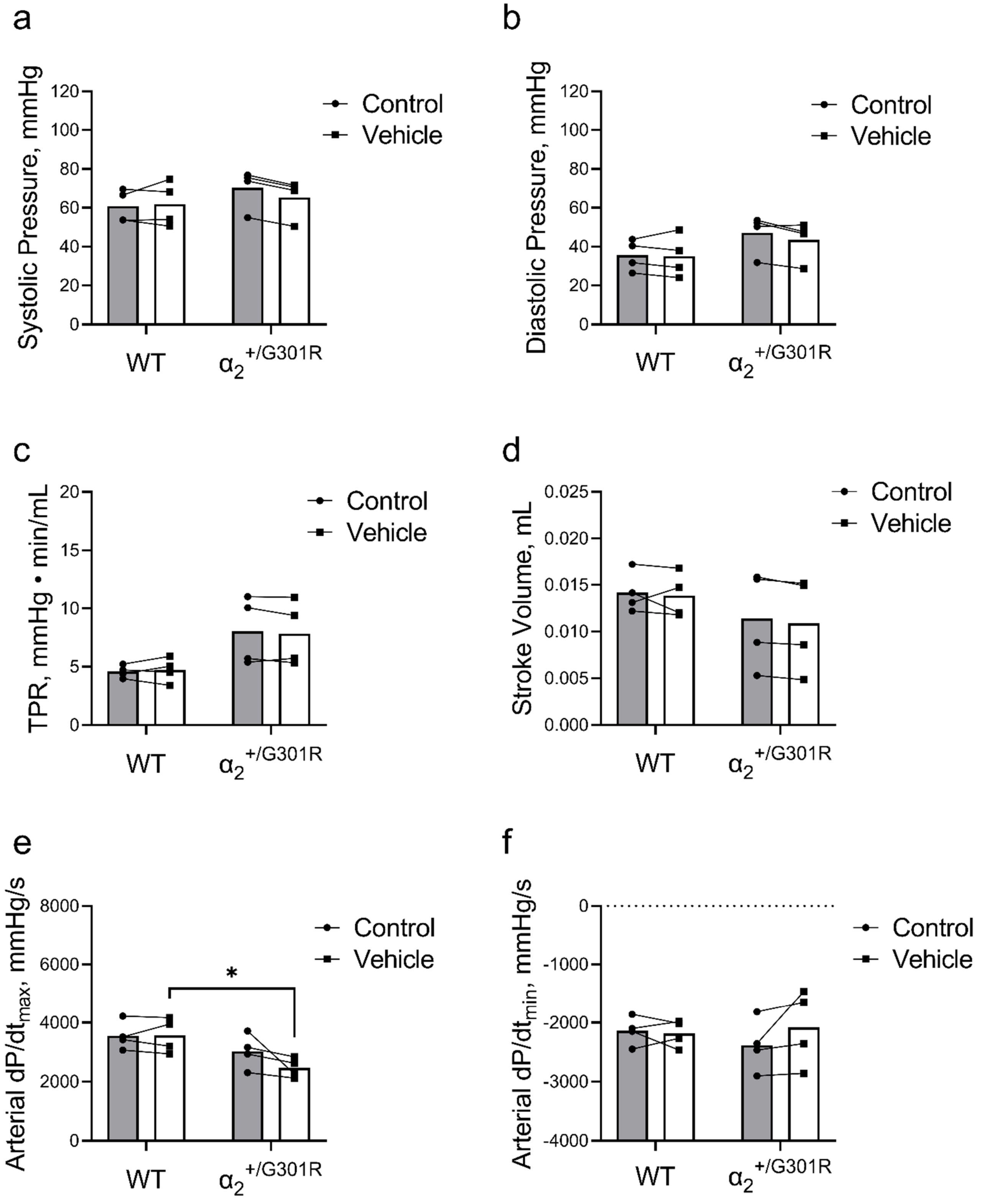

Figure S3. Cardiovascular variables were similar before and after vehicle (0.9% NaCl) administration during atrial pacing at 11.3 Hz (678 BPM) *in vivo*. (**a**) Systolic blood pressure (control vs. vehicle; 60.82 ± 4.22 mmHg vs. 61.86 ± 5.71 mmHg for WT and 70.25 ± 5.13 mmHg vs. 65.70 ± 5.01 mmHg for $\alpha_2^{+/G301R}$ mice), (**b**) diastolic blood pressure (control vs. ouabain; 35.62 ± 3.98 mmHg vs. 35.03 ± 5.39 mmHg for WT and 47.00 ± 5.09 mmHg vs. 43.53 ± 5.06 mmHg for $\alpha_2^{+/G301R}$ mice), (**c**) TPR (control vs. ouabain; 4.58 ± 0.26 mmHg · min/mL vs. 4.72 ± 0.52 mmHg · min/mL for WT and 8.04 ± 1.45 mmHg · min/mL vs. 7.85 ± 1.38 mmHg · min/mL for $\alpha_2^{+/G301R}$ mice), and (**d**) stroke volume (control vs. ouabain; 14.17 ± 1.09 µL vs. 13.83 ± 1.18 µL for WT and 11.40 ± 2.60 µL vs. 10.88 ± 2.52 µL for $\alpha_2^{+/G301R}$ mice) did not change after injection with vehicle when the heart was paced at 11.3 Hz. (**e**) Arterial dP/dt_{max} was greater in wild-type (WT) mice compared to $\alpha_2^{+/G301R}$ mice after vehicle treatment (WT vs. $\alpha_2^{+/G301R}$ mice; 3558 ± 241 mmHg/s vs. 3031 ± 290 mmHg/s under control conditions and 3567 ± 293 mmHg/s vs. 2467 ± 165 mmHg/s after vehicle injection; $P = 0.0192$). (**f**) Arterial dP/dt_{min} was similar between the two genotypes before and after vehicle administration (control vs. vehicle; -2138 ± 121 mmHg/s vs. -2182 ± 114 mmHg/s for WT and -2384 ± 224 mmHg/s vs. -2083 ± 322 mmHg/s for $\alpha_2^{+/G301R}$ mice). Data were compared with two-way repeated measures ANOVA followed by Bonferroni's multiple comparison test. $^*P < 0.05$; WT vs. $\alpha_2^{+/G301R}$ mice after vehicle administration. n = 4.

Supplementary Figure 4

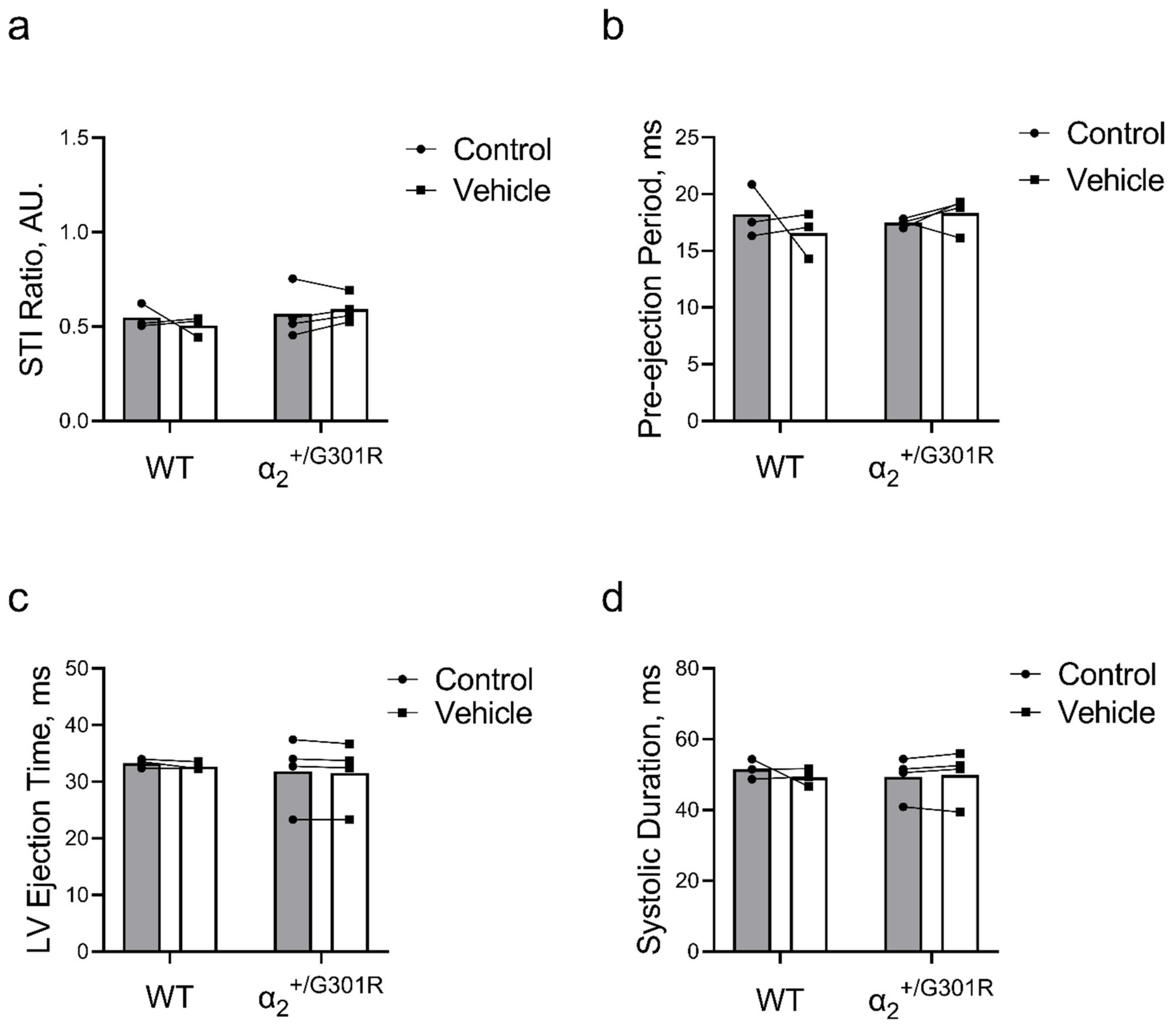

Figure S4. The systolic time intervals were unchanged after vehicle (0.9% NaCl) administration in $\alpha_2^{+/G301R}$ and wild-type (WT) mice during atrial pacing at 11.3 Hz (678 BPM). One WT mouse was excluded due to uninterpretable electrocardiogram recording, and the systolic time intervals were not calculated for this mouse. (**a**) The systolic time interval ratio (STI ratio; control vs. vehicle; 0.55 ± 0.04 vs. 0.51 ± 0.03 for WT and 0.57 ± 0.07 vs. 0.59 ± 0.04 for $\alpha_2^{+/G301R}$ mice), (**b**) pre-ejection period (control vs. vehicle; 18.3 ± 1.4 ms vs. 16.5 ± 1.2 ms for WT and 17.5 ± 0.2 ms vs. 18.4 ± 0.7 ms for $\alpha_2^{+/G301R}$ mice), (**c**) left ventricular ejection time (LV ejection time; control vs. vehicle; 33.3 ± 0.5 ms vs. 32.7 ± 0.4 ms for WT and 31.9 ± 3.0 ms vs. 31.5 ± 2.9 ms for $\alpha_2^{+/G301R}$ mice), and (**d**) systolic duration were unchanged after vehicle administration (control vs. vehicle; 51.5 ± 1.6 ms vs. 49.3 ± 1.5 ms for WT and 49.4 ± 2.9 ms vs. 49.9 ± 3.6 ms for $\alpha_2^{+/G301R}$ mice). Data were compared with two-way repeated measures ANOVA. *n* = 3-4.
