# Supplementary material for: Augmented Ouabain-Induced Vascular Response Reduces Cardiac Efficiency in Mice with Migraine-Associated Mutation in the Na+, K+-ATPase α2-Isoform
Source: Biomedicines. 2023 Jan 25;11(2):344. doi: 10.3390/biomedicines11020344 (PMC9953359; doi:10.3390/biomedicines11020344)
Supplement: Supplementary file 1 [file biomedicines-11-00344-s001.zip › biomedicines-2128057-supplementary-table.pdf]

Table S1. Changes to cardiovascular variables after i.p. injection of ouabain (1.5 mg/kg; also presented in Figure 2 and 3). At time = 0 minutes systolic blood pressure (79.63 ± 2.89 mmHg for WT and 71.31 ± 7.37 mmHg for $\alpha_2^{+/G301R}$ mice) and diastolic blood pressure (55.80 ± 2.77 mmHg for WT and 47.14 ± 7.65 mmHg for $\alpha_2^{+/G301R}$ mice) were similar between the two genotypes.

**Cardiovascular variables over time after i.p. injection of ouabain (1.5 mg/kg)**				
Minutes after injection	2 minutes	5 minutes	9 minutes	13 minutes
Systolic pressure (mmHg)				
Wild type	82.83 ± 2.92	86.67 ± 2.23	86.95 ± 2.89	89.83 ± 2.91
$\alpha_2^{+/G301R}$	75.44 ± 8.01	79.71 ± 7.52	80.98 ± 6.91	85.80 ± 8.06
Diastolic pressure (mmHg)				
Wild type	60.07 ± 2.85	63.84 ± 1.73	63.43 ± 2.06	65.16 ± 2.01
$\alpha_2^{+/G301R}$	50.91 ± 7.94	55.51 ± 7.33	56.88 ± 6.91	59.10 ± 7.42
ΔTotal peripheral resistance (mmHg• min/mL)				
Wild type	1.11 ± 0.39	1.57 ± 0.48	1.40 ± 0.42	1.07 ± 0.56
$\alpha_2^{+/G301R}$	0.40 ± 0.29	1.05 ± 0.30	1.70 ± 0.43	2.13 ± 0.47
ΔStroke volume (µL)				
Wild type	-0.63 ± 0.37	-0.28 ± 0.36	-0.01 ± 0.48	0.60 ± 0.76
$\alpha_2^{+/G301R}$	0.06 ± 0.33	0.06 ± 0.38	-0.52 ± 0.71	-0.53 ± 0.99
ΔHeart rate (BPM)				
Wild type	1 ± 7	-6 ± 4	14 ± 7	-5 ± 7
$\alpha_2^{+/G301R}$	2 ± 3	3 ± 4	-2 ± 8	-14 ± 14
ΔCardiac output (mL/min)				
Wild type	-0.33 ± 0.16	-0.25 ± 0.18	-0.21 ± 0.26	0.21 ± 0.45
$\alpha_2^{+/G301R}$	0.06 ± 0.17	0.06 ± 0.19	-0.34 ± 0.40	-0.59 ± 0.58
ΔSystolic time interval ratio (AU.)				
Wild type	0.02 ± 0.03	0.01 ± 0.03	0.00 ± 0.03	0.05 ± 0.04
$\alpha_2^{+/G301R}$	0.12 ± 0.04	0.14 ± 0.03	0.19 ± 0.05	0.19 ± 0.04
ΔPre-ejection period (ms)				
Wild type	0.5 ± 0.7	-0.0 ± 0.8	-0.4 ± 0.9	0.3 ± 1.2
$\alpha_2^{+/G301R}$	2.6 ± 0.8	3.2 ± 0.5	3.7 ± 1.0	3.6 ± 0.7
ΔLeft ventricular ejection time (ms)				
Wild type	-0.6 ± 0.3	-0.5 ± 0.6	-0.6 ± 0.6	-2.3 ± 1.2
$\alpha_2^{+/G301R}$	-1.9 ± 0.6	-2.5 ± 0.5	-3.7 ± 0.8	-3.7 ± 0.9
ΔSystolic duration (ms)				
Wild type	-0.2 ± 0.6	-0.5 ± 0.8	-1.1 ± 1.0	-2.0 ± 1.6
$\alpha_2^{+/G301R}$	0.7 ± 0.6	0.8 ± 0.5	0.1 ± 0.8	-0.2 ± 0.7
ΔArterial dP/dt_{max} (mmHg/s)				
Wild type	-226 ± 128	-206 ± 160	-83 ± 202	10 ± 240
$\alpha_2^{+/G301R}$	-16 ± 95	-30 ± 136	129 ± 227	228 ± 274
ΔArterial dP/dt_{min} (mmHg/s)				
Wild type	-8 ± 29	-44 ± 90	-103 ± 101	-181 ± 149
$\alpha_2^{+/G301R}$	-93 ± 52	-156 ± 70	-117 ± 99	-323 ± 181

Table S2. Changes to cardiovascular variables after i.p. injection of vehicle (NaCl 0.9%; also presented in Supplementary Figure 1 and 2. Just before i.p. injection of vehicle (0.9% NaCl; time = 0 minutes in Supplementary Figure 1 and 2), systolic blood pressure (64.32 ± 6.29 mmHg for WT and 70.06 ± 3.98 mmHg for $\alpha_2^{+/G301R}$ mice) and diastolic blood pressure (35.00 ± 5.37 mmHg for WT and 42.12 ± 5.26 mmHg for $\alpha_2^{+/G301R}$ mice) were similar between the two genotypes.

**Cardiovascular variables over time after i.p. injection of vehicle (0.9% NaCl)**				
Minutes after injection	2 minutes	5 minutes	9 minutes	13 minutes
Systolic pressure (mmHg)				
Wild type	66.47 ± 5.00	64.94 ± 6.48	67.00 ± 7.88	65.50 ± 10.63
$\alpha_2^{+/G301R}$	68.76 ± 2.40	68.82 ± 2.50	62.23 ± 4.51	69.19 ± 3.84
Diastolic pressure (mmHg)				
Wild type	37.40 ± 4.38	35.24 ± 6.08	35.52 ± 6.74	34.13 ± 9.22
$\alpha_2^{+/G301R}$	40.99 ± 3.75	40.90 ± 3.60	38.55 ± 7.08	42.48 ± 5.53
ΔTotal peripheral resistance (mmHg• min/mL)				
Wild type	0.03 ± 0.12	-0.13 ± 0.17	0.23 ± 0.28	0.18 ± 0.50
$\alpha_2^{+/G301R}$	0.15 ± 0.28	0.07 ± 0.26	-0.15 ± 0.24	-0.05 ± 0.11
ΔStroke volume (µL)				
Wild type	0.73 ± 0.41	0.59 ± 0.31	-0.88 ± 1.87	-0.91 ± 1.70
$\alpha_2^{+/G301R}$	-0.27 ± 0.22	-0.12 ± 0.23	-1.15 ± 0.62	-0.19 ± 0.26
ΔHeart rate (BPM)				
Wild type	3.91 ± 3.63	1.61 ± 4.65	12.17 ± 33.09	-5.04 ± 42.52
$\alpha_2^{+/G301R}$	-4.76 ± 2.65	-5.75 ± 5.31	-10.57 ± 10.97	3.92 ± 15.17
ΔCardiac output (mL/min)				
Wild type	0.48 ± 0.26	0.35 ± 0.19	-0.28 ± 0.51	-0.63 ± 0.27
$\alpha_2^{+/G301R}$	-0.18 ± 0.11	-0.13 ± 0.10	-0.61 ± 0.50	0.04 ± 0.16
ΔSystolic time interval ratio (AU.)				
Wild type	0.03 ± 0.06	0.02 ± 0.08	-0.05 ± 0.05	0.05 ± 0.01
$\alpha_2^{+/G301R}$	0.05 ± 0.08	-0.04 ± 0.08	-0.08 ± 0.05	-0.02 ± 0.05
ΔPre-ejection period (ms)				
Wild type	1.3 ± 2.2	0.6 ± 3.0	-2.2 ± 2.7	1.0 ± 1.6
$\alpha_2^{+/G301R}$	2.0 ± 3.5	-1.5 ± 3.3	-2.9 ± 1.8	-1.5 ± 1.9
ΔLeft ventricular ejection time (ms)				
Wild type	-0.1 ± 0.5	0.4 ± 0.3	-1.1 ± 1.5	-1.4 ± 2.5
$\alpha_2^{+/G301R}$	0.0 ± 0.3	0.7 ± 0.3	0.1 ± 1.0	-0.9 ± 0.9
ΔSystolic duration (ms)				
Wild type	1.2 ± 2.0	1.0 ± 2.9	-3.3 ± 4.2	-0.4 ± 4.1
$\alpha_2^{+/G301R}$	2.0 ± 3.6	-0.7 ± 3.2	-2.7 ± 1.8	-2.4 ± 1.7
ΔArterial dP/dt_{max} (mmHg/s)				
Wild type	-110 ± 162	-225 ± 139	-273 ± 299	-505 ± 134
$\alpha_2^{+/G301R}$	-292 ± 179	-346 ± 180	-975 ± 541	-395 ± 224
ΔArterial dP/dt_{min} (mmHg/s)				
Wild type	-47 ± 82	57 ± 131	-65 ± 188	75 ± 141
$\alpha_2^{+/G301R}$	113 ± 72	115 ± 68	540 ± 260	113 ± 48
